# Personal

**Published:** 1904-10

**Authors:** 


### PERSONAL.

DR. GROVER W. WENDE, of Buffalo, sailed for Europe in the last days of August to attend the International Congress of Dermatology, at Berlin, which was held in mid-September. Dr. Wende has already returned to Buffalo.

---

DR. CHARLES G. STOCKTON, 436 Franklin street, Buffalo, announces a change in his office hours. Hereafter, excepting Sundays, he can be found at home between the hours of ten and one o'clock.

---

Dr. Frank W. Low, of Buffalo, has removed his dental offices from his former location, No. 680 Main street, to No. 52 North Pearl street, between Virginia and Allen streets.

---

Dr. Eugene Smith, of Detroit, has been elected third vice-president of the American Academy of Ophthalmology and Oto-laryngology. This action was taken at the organisation's meeting in Denver, Col., August 27, 1904. It is possible that the next meeting may be held in Detroit.—*Detroit Med. Jour.*

---

Dr. J. Wesley Bovee, of Washington, D. C., has removed to The Rochambeau, Connecticut avenue and H street.

